# Identifying Soccer Teams’ Styles of Play: A Scoping and Critical Review

**DOI:** 10.3390/jfmk8020039

**Published:** 2023-03-30

**Authors:** Spyridon Plakias, Serafeim Moustakidis, Christos Kokkotis, Themistoklis Tsatalas, Marina Papalexi, Dionysios Plakias, Giannis Giakas, Dimitrios Tsaopoulos

**Affiliations:** 1 Department of Physical Education and Sport Science, University of Thessaly, Karyes, 42100 Trikala, Greece; 2 AIDEAS OÜ, Narva mnt 5, 10117 Tallinn, Estonia; 3 Department of Physical Education and Sport Science, Democritus University of Thrace, 69100 Komotini, Greece; 4 Department of Operations, Technology, Events and Hospitality Management, Manchester Metropolitan University, Oxford Road, Manchester M15 6BH, UK; 5 Network Rail, Piccadilly Tower, Manchester M60 7RA, UK; 6 Institute for Bio-Economy & Agri-Technology, Center for Research and Technology Hellas, 60361 Volos, Greece

**Keywords:** game style, playing styles, identify, contextual variables, effectiveness

## Abstract

Identifying and measuring soccer playing styles is a very important step toward a more effective performance analysis. Exploring the different game styles that a team can adopt to enable a great performance remains under-researched. To address this challenge and identify new directions in future research in the area, this paper conducted a critical review of 40 research articles that met specific criteria. Following the 22-item Preferred Reporting Items for Systematic Reviews and Meta-Analyses extension for Scoping Reviews (PRISMA-ScR) guidelines, this scoping review searched for literature on Google Scholar and Pub Med database. The descriptive and thematic analysis found that the objectives of the identified papers can be classified into three main categories (recognition and effectiveness of playing styles and contextual variables that affect them). Critically reviewing the studies, the paper concluded that: (i) factor analysis seems to be the best technique among inductive statistics; (ii) artificial intelligence (AI) opens new horizons in performance analysis, and (iii) there is a need for further research on the effectiveness of different playing styles, as well as on the impact of contextual variables on them.

## 1. Introduction

Performance analysis, i.e., the recording and examination of behavioral events occurring during a competition [1,2], is an essential tool in the hands of coaches. The relevant literature in soccer has traditionally focused on separated variables such as performance indicators to explain teams’ and players’ performance [3,4,5]. Performance indicators are variables that aim to define some or all aspects of performance. They may concern either a single action (e.g., pass, shot, recovery) or a combination of actions (ball possession, passes per defensive action, etc.) [6]. Recent research, in an attempt to analyze the complexity of the soccer game, has utilized playing styles instead of performance indicators, which may explain their tactical performance in matches and competitions more effectively [7,8].

Playing style is the characteristic pattern demonstrated and repeated by a team in specific situational contexts [9]. The scientific literature around playing styles has grown significantly, with some authors defining game styles based on their subjective perceptions. Notably, various styles have been mentioned such as supported, direct, defensive pressing, attacking [10], very direct and aggressive [11], direct, possession, offensive [12], Dutch [13], positional play [14], FC Barcelona style [15], and more.

The most remarkable shift in football’s evolution is the application of scientific tools supported by scientific data [16]. The use of event statistical data (from new software tools such as Sportscode, Nacsport, and Longomatch) and tracking data (from optical methods and GPS) has ushered in the Big Data era [17]. The intersection of data science and sport science has tried to unlock the potential of big data to support tactical performance analysis with various methods, such as AI, multivariate statistical techniques and visualizations [18,19,20]. Researchers have successfully used AI techniques to address soccer analytics tasks such as detecting tactics [21]. In the field of game style identification, the multivariate statistical methods that are mostly used include factor analysis accompanied with principal component analysis (PCA), and k-means clustering. When the research question concerns the effect of contextual variables or the effectiveness of playing styles, then MANOVA and MANCOVA (Multivariate Analysis Of Variance and Covariance) are usually employed. Contextual variables refer to factors that can potentially affect performance, such as match status, match location, opponent level, type of competition, period of the season, playing surface, etc. [22]. Lastly, many studies have employed visualizations, transforming non-visual quantified data into visual forms, to facilitate a better comprehension of the results [23].

According to the above: (i) it is a given that the concept of “playing style” is used to better understand the complexity of the game and team tactics, (ii) there is a growing interest from researchers in recent years on playing styles, and (iii) there is not a single way of using the term, while many times it is used without scientific validation. Furthermore, despite researchers’ efforts, there are still several knowledge gaps in relation to expert interviews during qualitative studies, and so far, no attempt has been made in the literature to review the subject comprehensively. Therefore, the objectives of this review are: (i) to critically examine how the term is used in the international literature, how research is conducted on this topic and what are the main objectives of the research, and (ii) to identify and analyze knowledge gaps in order to contribute to future research and a future systematic review. By conducting this comprehensive analysis, we hope to provide valuable insights for researchers, coaches, and practitioners, enhancing their understanding of soccer playing styles and their effectiveness in improving team performance.

## 2. Materials and Methods

This scoping review followed the 22-item Preferred Reporting Items for Systematic Reviews and Meta-Analyses extension for Scoping Reviews (PRISMA-ScR) [24]. Furthermore, to conduct the critical review, this paper has critically reviewed the selected articles, following the process adopted by Mackenzie and Cushion [25].

### 2.1. Literature Search Approach

This review was based on research articles published up until 25 September 2022 (with no restriction on the year of publication) using the search engines PubMed, Web of Science, and Google Scholar. During our search in PubMed and Web of Science, we combined the terms “soccer” and “style” using the Boolean operator AND. In contrast, when searching Google Scholar, due to the large number of initial results, we utilized the expression “soccer style” as a more focused search term. A prerequisite for the inclusion of an article in our study was the presence of the two mentioned terms (in singular or plural) as keywords, either in the title or in the abstract of each article [26].

### 2.2. Exclusion Criteria

Initially, two of the authors (S.P. and C.K.) checked the titles and abstracts of all retrieved publications. In cases where a disagreement arose between the two reviewers during the article selection process, a third independent reviewer was employed to resolve the discrepancy and reach a consensus. The following categories were excluded: (i) non-English articles, (ii) postgraduate and doctoral dissertations, review articles and books (iii) studies of styles in other sports, (iv) research based on robotic soccer and video games (v) articles related to individual player, coach or referee styles, and (vi) studies that did not focus on playing styles. All the selected articles are peer-reviewed and have been presented either in journal papers or conferences. Finally, the rest of the authors reviewed again the titles and abstracts to ensure that they met the inclusion criteria.

### 2.3. Assessed Outcomes

The studies, which are recorded in this article, were divided into three categories, namely: (i) recognition of playing styles, (ii) contextual variables that influence the adoption of each style, and (iii) effectiveness of styles. The grouping was based on the most common objectives of the studies. Articles investigating multiple concepts were included in more than one category [27]. The first category includes all articles with the aim of separating distinct styles, identifying styles’ characteristics and quantifying them. Then, after separating the articles, the following information was extracted from each article: author, year of publication, method (classical inductive statistics/A.I./other), sample (number of matches, competitions), kind of data (P.I./tracking data/other), phases of the game concerning the styles studied, outcome.

## 3. Results

### 3.1. Search Results

**Figure 1:**
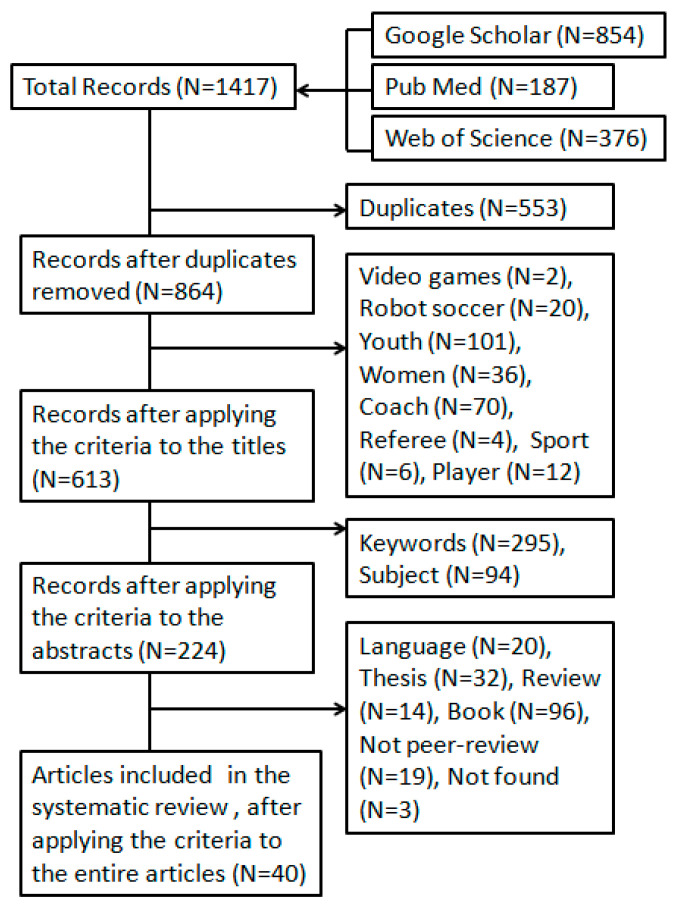
Flow chart of the methodology used for the article search.

The initial search identified 1417 titles in the described databases. After duplicates were removed the remaining 864 articles were then screened for relevance based on their title, resulting in another 251 studies being eliminated from the database. The abstract of the remaining 613 articles was then read and another 389 were rejected due to a lack of relevance to the purpose of this study. Finally, only 40 articles remained for the scoping review, when the entire articles were read (Figure 1).

### 3.2. Descriptive Analysis

**Figure 2:**
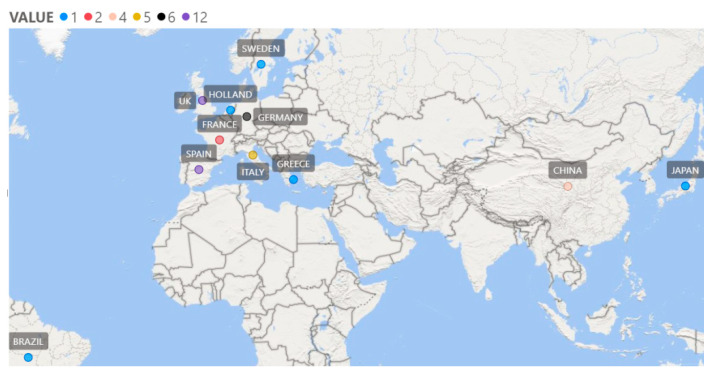
Map of countries whose leagues have been included in relevant surveys (the colour of the bubble represents the number of studies in which they have been included).

A total of 40 articles were identified in the review, after applying the proposed inclusion/exclusion criteria. In total, 29 of the 40 articles used classical inductive statistics alone or in combination with other methods (e.g., visualization), 10 used AI methods alone or in combination with other methods (e.g., visualization), and one study combined classical statistics with A.I. and observational methodology (Table 1). Table 1 also shows that 24 surveys used performance indicators from event data, eight used tracking data, while the rest used other types of data (flow motifs, team possessions, questionnaires, etc.). In the same table it can be seen that the sample was usually taken from country leagues (mainly the big four European i.e., English, Spanish, German, Italian) and the World Cup. The map in Figure 2 illustrates the domestic competitions that have been included in the relevant surveys.

### 3.3. Thematic Analysis

**Figure 3:**
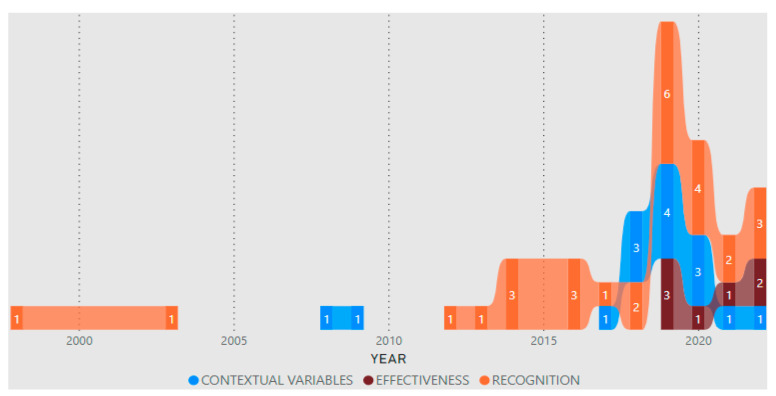
Number of articles by year and category.

Table 2 shows the separation of studies based on the main categories mentioned in the methodology (assessed outcomes), considering that some of the articles belong to more than one category. Figure 3 provides a visualization of the number of articles by year and category.

## 4. Discussion

### 4.1. Recognition

The vast majority of related literature has focused on game style recognition. Historically, explanations for the performance of teams and individual players were based on singular events or isolated behaviors. Basevitch, Yang and Tenenbaum [4], and Castellano and Echeazarra [42] tried to match separate variables with the playing style of teams. Then, they tried to identify variations on the specific variables between the teams. Despite the significance of the information offered by these studies, they cannot cover the complexity of the soccer game, which requires the connection of different performance indicators for recognizing a team’s styles of play or tactical pattern [7,8]. To bridge this gap, Kempe, Vogelbein, Memmert, and Nopp [32], introduced two indexes to differentiate between possession play and direct style. Each index was the sum of z-values of performance indicators considered by the authors to determine game control and offensive behavior. The research was limited by the absence of validation for the two indexes, as the variables employed in their formulation were selected based on the authors’ subjective perceptions. K-means cluster analysis was also used by Gollan, Ferrar, and Norton [36] to recognize playing styles. Three game style clusters were identified: (1) moderately favoring established defense, (2) dominant in transition offense and transition defense, and (3) strong in established offense and set pieces. The disadvantage of this method is that it does neither recognize playing styles, nor is it capable of quantifying them; instead, it categorizes the teams based on the phases in which they excel. Finally, the studies of Drezner, Lamas, Farias, Barrera and Dantas [43], Fernandes, Camerino, Garganta, Hileno and Barreira [46], and Tenga and Larsen [29] relied on the construction of their own models for game style recognition. The first two divided the field into sections (18 and 14, respectively). After recording the sequences of passes throughout each ball possession (from the beginning to its completion), Chi-Square analysis was performed. The first research found different styles between teams for ball circulation, while the second focused on defensive behavior patterns. Due to the time-consuming aspect of studying all ball possession sequences, it is difficult for the aforementioned studies to investigate a large sample of matches. Thus, nine matches were analyzed in the first and 12 in the second. In a match between Norway and Brazil, Tenga and Larsen [29] utilized a similar technique involving the development of their own model and Chi-Square analysis. However, they did not divide the field into zones.

**Figure 4:**
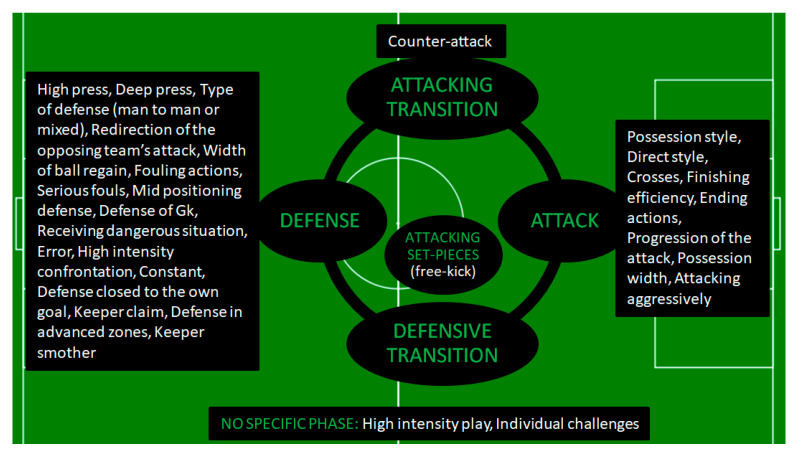
Classification of playing styles identified by Factor-PCA in each of the game phases.

Among all studies that applied inductive statistics, those that used factor analysis with PCA (Table 3) were the ones that managed not only to distinguish distinct styles, but also to identify the characteristics of each style (based on the variables that loaded each component). In addition, they were able to quantify playing styles based on the factor scores. Nonetheless, as shown in Table 3 and Figure 4, the styles of play identified in published research have not been able to account for all phases and sub-phases of the game. For example, the absence of defensive set pieces can be seen. Despite the fact that, in the research of Gómez, Mitrotasios, Armatas, and Lago-Peñas [8], two factors have been given the names set pieces and free kicks, it is clear (from observing the variables that load them) that they concern the attacking set-pieces and the attacking free-kicks, respectively. This subjectivity in the naming of latent variables is a disadvantage of studies using factor analysis [64]. Factors may not have been correctly labeled, and it should not be assumed that two factors with the same name are the same thing (jingle fallacy) [65]. For example, in the research of Lago-Peñas, Gómez-Ruano, and Yang [34], factors 4 and 5 are given the general name transitional play. However, observing the variables that load the factors, we find that the variables of factor four (lost balls) can only appear in situations of defensive transition, while the variables of factor five (picking up free balls) can only appear in situations of offensive transition. Similarly, Ruan, Ge, Gómez, Shen, Gong, and Cui [50] give factors 6 and 8 the same name (defense of goalkeepers) even though, as the authors report, different teams have higher values in each of them. Moreover, in the study by Gómez, Mitrotasios, Armatas and Lago-Peñas [8], factor four was named counter-attack despite the fact that one of the loaded variables is lost balls, which cannot result in a counter-attack. So, either it should be clarified that the style concerns the game as a whole (for both teams together) or if the authors wish to adopt it as a single team’s style, a more general label (such as transitional play) should be given, which can cover both offensive and defensive transitions. In the same study, factor six is exclusively related to the variable actions in own fourth (they had divided the field into fourths along its length and by own fourth they meant the one near the team’s goal). However, from what has come to our attention, it is unclear why this particular factor was called transitional play and more explanation should be provided by the authors. 

Therefore, although factor analysis is an excellent solution for grouping variables to construct playing styles, special care is required when interpreting and naming components based on the variables that load on them. If this obstacle can be cleared away, then factor analysis could be considered as a valuable tool that offers useful information in distinguishing, identifying and quantifying playing styles. Many playing styles, such as those that concern the first sub-phase of the attack (the “build up from the back”) and others, have yet to be identified in a factor-based study. As examples, we mention the offside trap, which is a very common tactical tool of coaches [66,67,68,69], the passing tempo that gives very useful information about the playing style of a team [12,40,70], the counter-pressing [71,72] and so on. Further, only two studies employing factor analysis with PCA [47,50] had access to physical performance variables. However, they found no association between these variables and the styles emerging from technical-tactical variables. Lastly, despite the fact that nearly all of the aforementioned research referred to phases of the game, none of them gave a structured model classifying the detected styles in the various key moments and sub-phases.

The use of artificial intelligence methods in studies related to the playing styles of teams in soccer has given new dimensions to the research on this specific topic. The first to adopt a simplistic form of artificial intelligence for game style recognition were Gyarmati, Kwak, and Rodriguez [52], who used two clustering techniques (K-means and Ward hierarchical). Both methods showed that Barcelona had a unique style (based on its passing motifs) compared to all the other teams. The same year, Bialkowski, Lucey, Carr, Yue, Sridharan, and Matthews [53], employed K-means clustering and Linear Discriminant Analysis (LDA) and distinguished five different styles adopted by teams during matches. Two years later, the same author published a similar article in which agglomerative clustering was also utilized [54]. Brooks, Kerr, and Guttag [55] divided the field into 16 zones and counted the number of passes that originated from each zone. The classification task was then accomplished using K-nearest neighbor (K-NN) classifier and a heatmap of pass origins. The field has also been divided into zones (20) by Amatria, Maneiro and Anguera [62]. They recorded the origin and destination of each pass in each distinct sequence of ball possession. At the same time, technical actions (ball control, dribbling) were recorded. Then they applied T-pattern detection to identify patterns of play in possession. Finally, Bekkers and Dabadghao [56] used a mean shift algorithm to cluster teams based on their passing motifs, where four clusters emerged. 

Narizuka and Yamazaki [57] adopted the Delaunay network to get (as adjacency matrix A(t)) the formation of a team at time t. Then, using hierarchical clustering, they obtained not only the average formation (i.e., “442”, “4141”, “433”, “541” or “343”) for each team in the match but also the positional exchange of players within the match formations. Decroos, Roy, and Davis [58] used mixture models to achieve a representation of soccer actions. In the first stage, for each action type, a mixture model was fitted to the locations (x, y). Then, for each component of each mixture model in stage 1, a new mixture model was fitted to the directions of the actions in that component. Using the learned mixture models, each action was encoded as a weight vector. It was concluded that using specific vectors, game styles could be identified based on each type of action. Finally, García-Aliaga, Marquina Nieto, Coterón, Rodríguez-González, Gil Ares, and Refoyo Román [60] employed the t-distributed Stochastic Neighbor Embedding (t-SNE) clustering algorithm, which can process large multivariate datasets and visualize them into a 2D plot [73]. Consequently, the various placements of the teams in the plots reflect the disparity between their playing styles.

As demonstrated above, a small number of studies focused solely on the possession phase of the ball [52,55,56,62]. The studies of Bialkowski, Lucey, Carr, Yue, Sridharan, and Matthews [53], Bialkowski, Lucey, Carr, Matthews, Sridharan. and Fookes [54], and Narizuka and Yamazaki [57] reached only conclusions regarding team formations. Bialkowski, Lucey, Carr, Yue, Sridharan, and Matthews [53] and Bialkowski, Lucey, Carr, Matthews, Sridharan, and Fookes [54] distinguished five different styles of play, however without information about the characteristics of each style, they are of limited practical value to coaches. After classifying the teams, García-Aliaga, Marquina Nieto, Coterón, Rodríguez-González, Gil Ares, and Refoyo Román [60], used the RIPPER method to find the variables that contribute most to the categorization of the teams. This has practical value, as opposed to simply grouping things together. The vectors of the actions which were created in the research of Decroos, Roy, and Davis [58] gave more information than simple numbers, but they also refer to separate variables. However, the ability to examine them simultaneously can provide valuable information to the coaching staff.

### 4.2. Contextual Variables

**Figure 5:**
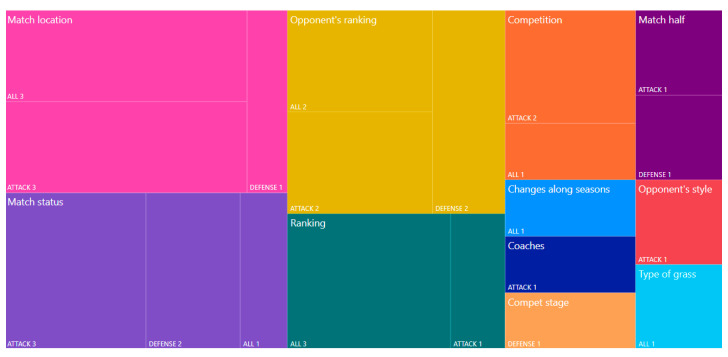
Proportion of articles based on contextual variables and game phases in which their effect on teams’ playing styles was studied.

Contextual variables (specific match factors) have an impact on competitive demands and influence performance metrics [74,75]. In recent years, there has been a strong tendency to study the effect of contextual variables on the teams’ adoption of different playing styles. As shown in Table 2 and Figure 3, twelve of the thirteen relevant studies were conducted from 2018 onwards. The Treemap in Figure 5 shows that the majority of studies have explored the effect of match location. Particularly, Lago [30], Santos, Lago-Peñas, and García-García [35], Gómez, Mitrotasios, Armatas, and Lago-Peñas [8], Fernandez-Navarro, Fradua, Zubillaga, and McRobert [37], Bekkers and Dabadghao [56], Gollan, Bellenger, and Norton [44], and Gonzalez-Rodenas, Aranda, and Aranda-Malaves [45], dealt with the impact of the variable match location on the playing styles adopted by the teams. From all the above studies it seemed that home teams tried to build up from the back, rather than adopt direct play, resulting in a more combinational, possession-based style of play. They also seemed to try to speed up the match and press their opponents high, whereas in general, their game was more offensive.

Team’s ranking, opponent’s ranking, and match status are also variables whose effect on playing styles has been studied extensively. The research of Gonzalez-Rodenas, Aranda, and Aranda-Malaves [45], showed that high-ranked teams were less likely to use direct attacks than combinative attacks in comparison with low-ranked teams. García-Aliaga, Marquina Nieto, Coterón, Rodríguez-González, Gil Ares, and Refoyo Román [60] found that the top-ranked teams in the EPL differentiated from the rest of the teams in the same league by making less long passes, playing more vertically, even at the expense of accuracy, and dribbling. In [44], using EPL data of the 2015–2016 season in which Leicester shocked the league by winning it, it was demonstrated that established defense was prevalent among lower-ranked teams, while the champion was dominant in transitions and the rest of the high-ranked teams dominated established attack and set pieces. In this case, there appears to be a disadvantage associated with studies that collect their sample data from a single competition or, even worse, from a small number of matches. In such cases, generalizing the outcomes is inherently risky, as teams like Leicester, for instance, rarely win the league. As shown in Table 1, only 13 of the 40 studies processed data from more than one competition. As demonstrated by Lago [30], Santos, Lago-Peñas, and García-García [35], Fernandes, Camerino, Garganta, Hileno, and Barreira [46], Gollan, Bellenger, and Norton [44], and Gonzalez-Rodenas, Aranda, and Aranda-Malaves [45], the quality of the opposition exhibited similar trends with math location. Finally, match status appears to influence the adoption of different playing styles significantly. In particular, winning teams present higher probabilities of attacking by means of counterattacks and direct attacks than combinative attacks compared to losing teams. In addition, they apply high pressure less frequently [37,41,45,46].

However, there are some contextual variables whose effect on playing styles has received little or no attention. Comparisons between different competitions were performed amongst the top four leagues in Europe according to the UEFA ranking (English, Spanish, German, and Italian). Although Gonzalez-Rodenas, Aranda, and Aranda-Malaves [45] found no statistically significant differences between the English and Spanish leagues during the 2017–2018 season, Mitrotasios, Gonzalez-Rodenas, Armatas, and Aranda [40] discovered that Spain La Liga had a higher proportion of long and combinative attacks, the English Premier League had a higher tendency of progressing through fast and direct attacks, the German Bundesliga had the most counter-attacks, and the Italian Serie A had the shortest offensive sequences as well as a higher proportion of counter-attacks and direct attacks than combinative and fast attacks. García-Aliaga, Marquina Nieto, Coterón, Rodríguez-González, Gil Ares, and Refoyo Román [60] found a distinction between the English teams and the rest of the teams in the other leagues, determined by fewer free kicks, fewer long passes but more vertical, more errors in ball control, and greater success in dribbling, using data from the specific four countries from five different seasons (from 2014/2015 to 2018/2019).

The effect of match half time (first or second) was examined in two studies. One for the attacking phase [45] and one for the defensive phase [46]. The first found that progressing by counterattacks was less likely in the first half than progressing with combinative attacks in the second half. In the latter, the national teams of Germany and Argentina displayed defensive tactics in the second half that did not exist in full matches. The effect of five contextual variables on the playing styles (type of grass, stage of the competition, coaches, changes along years, and opponent’s style) was explored in just one study for each variable. Andersson, Ekblom, and Krustrup [3] observed fewer sliding tackles and more short passes during games on artificial turf as compared to natural grass. According to Yi, Gómez, Wang, Huang, Zhang, and Liu [63], teams performed better in passing, pass accuracy, and delivery into the attacking third when playing against direct-play teams than when playing against possession-play teams. Bekkers and Dabadghao [56] found that when teams change managers, their flow motifs change, adopting the style of the specific manager’s prior teams. Fernandes, Camerino, Garganta, Hileno, and Barreira [46] found that some teams may change defensive patterns along stages. Finally, Zhou, Lago-Peñas, Lorenzo, and Gómez [47] found that high-intensity play and offensive actions of Chinese SL increased substantially with time (from 2012 to 2017). It is impressive that the effect of formation on the playing style of teams has not yet been studied, despite that fact that it is an important element of their tactics [76], while the scientific literature has already shown that it affects physical and technical performance indicators [77,78]. The effect of other contextual variables could also be studied, such as the period of the season [79] or the market value of the teams [80].

### 4.3. Effectiveness

Seven papers studied the effectiveness of playing styles. Of particular interest is the distinct way in which each study expressed efficacy. Four of the studies that measured effectiveness solely addressed styles during the attacking phase. Fernandez-Navarro, Fradua, Zubillaga, and McRobert [20], used the possession effectiveness index which is a combination of the variables Expected Goals (shot location and shot type were the variables considered to calculate this metric) and Ball Movement Points that measures each ball move in a possession. Ball moves were assessed based on the risk they pose to the opponent. The results suggested that the effectiveness of game styles changes under specific circumstances and that not all contextual variables affect them in the same way. Bekkers and Dabadghao [56] measured the effectiveness of styles of play by introducing an Expected Goal Motifs model (a sequence of at least one pass that leads to a goal-scoring opportunity with a certain expectation of being converted). Drezner, Lamas, Farias, Barrera, and Dantas [43] defined two main classes-incomplete and complete penetration dynamics. Incomplete penetration dynamics were defined as those that do not reach the opponent’s last defensive line. As a result, the effectiveness was calculated according to the degree of success of the ball circulation that leads to a penetration. Finally, in Schulze, Julian, and Meyer [49] study, goals and goal scoring opportunities (GSOs) were used to measure the effectiveness of attacks. In the two papers that examined all phases of the game, Castellano and Pic [39] based their study on the final result (win/lose/draw), while Lopez-Valenciano, Garcia-Gómez, López-Del Campo, Resta, Moreno-Perez, Blanco-Pita, Valés-Vázquez, and Del Coso [7] based their study on the number of points obtained and the final ranking position. Only one study has dealt with the effectiveness of defensive playing styles, for which the expected goals of the opponent were used to calculate [51]. A positive aspect of the studies of Castellano and Pic [39] and Lopez-Valenciano, Garcia-Gómez, López-Del Campo, Resta, Moreno-Perez, Blanco-Pita, Valés-Vázquez, and Del Coso [7] is that they did not merely use the data of the provider companies (e.g., InStat, Tracab, Mediacoach), but instead they created new variables (from the already existing ones), taking into account the interaction between the two teams or by applying normalizations of the data based on the time of possession, the number of attacks, etc.

## 5. Conclusions

This article provides a critical analysis of the research that relates to the playing styles of soccer teams and meets specific criteria. It was found that the data from a single competition is insufficient to generalize the outcomes of the studies. Researchers should place a greater emphasis on the practicability of their findings for team coaches. Visualization of conclusions is a method that helps in this direction. In addition, the utilization of extra factors and, in particular, the invention of new ones (with more tactical significance) will assist in identifying alternative play styles and evaluating their performance. AI and factor analysis can provide useful information in the identification of playing styles, but the latter requires attention to the interpretation of the components. More research on playing styles that focuses on the actual application of findings on football fields is therefore required.

## Figures and Tables

**Table 1:** The method, the kind of data, and the sample (matches) used in the articles.

Author	Year	Category	Method	Kind of Data			Sample (Number of Matches & Competitions)	
				P.I. (Event)	Tracking Data	Other		
Pollard [28]	1998	CLASSICAL INDUCTIVE STATISTIC (C.I.S.)	Factor-PCA	*			74	1982 World Cup, English Premier League (EPL) 1984–85
Tenga [29]	2003		Chi-square	*			1	Brazil-Norway
Andersson [3]	2008		Two-way ANOVA, paired *t*-test, chi-square	*	*	Questionaries	10	Male/female Swedish league
Lago [30]	2009		Linear regression	*			25	Spanish league 2005–2006
Sporiš [31]	2012		Factor-PCA, Cronbach’s alpha	*		Rating from 0–5 from ten experts	_	_
Basevitch [4]	2013		Independent *t*-tests. Multiple linear regression	*			_	Brazilian and Italian matches from all the World Cups, Brazilian and Italian premier leagues from 2003 to 2008
Kempe [32]	2014		ANOVA	*			676	Bundesliga 2009/10 & 2010/11, FIFA World Cup 2010
Fernandez-Navarro [33]	2016		Factor-PCA	*			97	Spanish La Liga and EPL from the seasons 2006–2007 and 2010–2011
Lago-Peñas [34]	2017		Factor-PCA	*			240	Chinese Super League (SL) during the 2016 season
Santos [35]	2017		Linear regression		*	Ball recovery situations	13	An elite Spanish team
Gómez [8]	2018		Factor-PCA, ANCOVA, MANOVA	*			301	Greek SL during the 2013–2014 regular season
Gollan [36]	2018		K-means clustering, chi-square	*			380	2015–16 EPL
Fernandez-Navarro [37]	2018		Linear mixed model (cross-classified multilevel design)			Each possession	380	2015–16 EPL
Yi [38]	2019		k-means clustering, Separate Poisson regression models	*			59	2018 FIFA World Cup
Fernandez-Navarro [20]	2019		linear mixed model (cross-classified multilevel design)			Possession Effectiveness Index	380	2015–16 English Premier League
Castellano [39]	2019		Factor-PCA, Discriminant analyses, Chi-square			Subtraction between the P.I. value of one team and the P.I. of the other	373	2016–2017 season of the Spanish first division (LaLiga)
Mitrotasios [40]	2019		Kruskal–Wallis, Mann–Whitney			Team possessions	80	Spanish, English, German and Italian first division during 2017–2018 season
Praça [41]	2019		Social network analysis, one-way ANOVA, two-way ANOVA			Passes	14	2018 FIFA World Cup
Castellano [42]	2019		Social network analysis, magnitude-based inference and correlation		*	Passes	36	La Liga 2017/18
Drezner [43]	2020		Chi-square			Characteristics of ball possessions	9	Champions League
Gollan [44]	2020		Odds ratios, logistic regression analysis	*			380	2015-16 EPL
Gonzalez-Rodenas [45]	2020		Multivariate logistic regressions			Team possessions	40	Spanish La Liga and EPL 2017–2018
Fernandes [46]	2020		Kruskal–Wallis H, Mann–Whitney U, Chi-square, Z-, multinomial logistic regression, sequential analysis	*			12	2014 FIFA World Cup
Zhou [47]	2021		Factor-PCA, MANCOVA	*			1429	Chinese SL matches from 2012 to 2017
Amatria [48]	2021		Pearson’s chi-square statistic	*			39	2016–2017, 2017–2018, and 2018–2019 Champions League
Schulze [49]	2021		Factor-PCA, Linear regressions		*		one team’s games	German 2016/2017 season
Lopez-Valenciano [7]	2022		Pearson’s correlation coefficient tests, Spearman’s rank correlation coefficient test and PCA	*			760	2017–2018 and 2018–2019 seasons of the Spanish national league
Ruan [50]	2022		Factor-PCA	*	*		240	Chinese Super League 2018
Ruan [51]	2022		PCA, multivariate regression model	*			1120	Chinese Super League 2016 –2020
Gyarmati [52]	2014	AI	K-means clustering, Ward hierarchical clustering			Flow motifs	all	2012/13 season of the Spanish, Italian, English, French, and German first division
Bialkowski [53]	2014		K-means clustering, LDA, k-NN regression	*	*		374	One league
Bialkowski [54]	2016		k-means clustering, agglomerative clustering, linear discriminant analysis, k-nearest neighbour	*	*		374	one league
Brooks [55]	2016		K-nearest neighbor, L2-regularized support vector machine model			Every pass (with 8 descriptors)	_	2012–2013 La Liga
Bekkers [56]	2019		Mean shift algorithm			Flow motifs	8219	4 seasons (2012/2013 to 2015/2016), 6 different leagues (Dutch, English, Spanish, Italian, French and German first division)
Narizuka [57]	2019		Extended clustering algorithm based on role representation (and hierarchical clustering)		*		45	Japanese league 2016
Decroos [58]	2020		Mixture models			Actions described by their type, location, and direction	760	2017/18 and 2018/19 seasons of the EPL
Beernaerts [59]	2020		Qualitative Trajectory Calculus		*		1	2016–2017 professional soccer competition
García-Aliaga [60]	2022		t-SNE dimensionality reduction technique, classification rules with RIPPER	*			all	EPL, Spanish LaLiga, German Bundesliga, and Italian Serie A from the 2014/2015 to 2018/2019 seasons
Lee [61]	2022		Deep Neural Networks (DNN) based on Multi-Layer Perceptron (MLP) and feature engineering	*			(a) all Tottenham’s games, (b) 380	(a) 11 seasons (2010/2011–2020/2021) English premier league, (b) 2020-21 EPL
Amatria [62]	2019	C.I.S. & AI	Cohen’s kappa & T-pattern analysis			Team possessions	7	UEFA Euro 2012

**Table 2:** Distinction of articles into three categories based on their main purpose.

Recognition (28)	Contextual Variables (15)	Effectiveness (7)
Pollard, Reep and Hartley [28], Tenga and Larsen [29], Sporiš, Šamija, Vlahović, Milanović, Barišić, Bonacin and Talović [31], Basevitch, Yang and Tenenbaum [4], Gyarmati, Kwak and Rodriguez [52], Bialkowski, Lucey, Carr, Yue, Sridharan and Matthews [53], Kempe, Vogelbein, Memmert and Nopp [32], Fernandez-Navarro, Fradua, Zubillaga, Ford and McRobert [33], Bialkowski, Lucey, Carr, Matthews, Sridharan and Fookes [54], Brooks, Kerr and Guttag [55], Lago-Peñas, Gómez-Ruano and Yang [34], Gómez, Mitrotasios, Armatas and Lago-Peñas [8], Gollan, Ferrar and Norton [36], Bekkers and Dabadghao [56], Castellano and Pic [39], Amatria, Maneiro and Anguera [62], Narizuka and Yamazaki [57], Praça, Lima, Bredt, Sousa, Clemente and Andrade [41], Castellano and Echeazarra [42], Drezner, Lamas, Farias, Barrera and Dantas [43], Decroos, Roy and Davis [58], Fernandes, Camerino, Garganta, Hileno and Barreira [46], Beernaerts, De Baets, Lenoir and Van de Weghe [59], Zhou, Lago-Peñas, Lorenzo and Gómez [47], Amatria, Maneiro, Casal, Papadopoulou, Sarmento, Ardá, Iglesias and Losada [48], García-Aliaga, Marquina Nieto, Coterón, Rodríguez-González, Gil Ares and Refoyo Román [60], Ruan, Ge, Gómez, Shen, Gong and Cui [50], Ruan, Ge, Shen, Pu, Zong and Cui [51]	Andersson, Ekblom and Krustrup [3] Gómez, Mitrotasios, Armatas and Lago-Peñas [8], Gollan, Ferrar and Norton [36], Fernandez-Navarro, Fradua, Zubillaga and McRobert [37], Mitrotasios, Gonzalez-Rodenas, Armatas and Aranda [40], Praça, Lima, Bredt, Sousa, Clemente and Andrade [41], Yi, et al. [63], Bekkers and Dabadghao [56], Gollan, Bellenger and Norton [44], Gonzalez-Rodenas, Aranda and Aranda-Malaves [45], Fernandes, Camerino, Garganta, Hileno and Barreira [46], Zhou, Lago-Peñas, Lorenzo and Gómez [47], García-Aliaga, Marquina Nieto, Coterón, Rodríguez-González, Gil Ares and Refoyo Román [60], Santos, Lago-Peñas and García-García [35], Lago [30]	Fernandez-Navarro, Fradua, Zubillaga and McRobert [20], Bekkers and Dabadghao [56], Castellano and Pic [39], Drezner, Lamas, Farias, Barrera and Dantas [43], Schulze, Julian and Meyer [49], Lopez-Valenciano, Garcia-Gómez, López-Del Campo, Resta, Moreno-Perez, Blanco-Pita, Valés-Vázquez and Del Coso [7], Ruan, Ge, Shen, Pu, Zong and Cui [51]

**Table 3:** Factors extracted in the studies employing factor analysis with PCA.

Article	Factors’ Names
[28]	Possession style, Crosses, High press
[31]	Finishing efficiency, Ball possession performance, Counter-attack efficiency, Type of defense (man to man to man/ mixed), Redirection of the opposing team’s attack build-up
[33]	Possession directness, Width of ball regain, Use of crosses, Possession width, Defensive ball pressure, Progression of the attack
[34]	Possession style, Set pieces attack, Counterattacking play, Transitional play (2) *
[8]	Ball possession, Ending actions, Individual challenges, Counter attack, Set pieces, Transitional play, Fouling actions, Free-kick
[39]	High press, type of attack
[47]	High intensity play, Possession and passing, Offensive actions, Defensive actions, Individual challenges, Serious fouls, Attacking aggressively
[50]	Defense close to the own goal, High intensity confrontation, Mid positioning defense with pressure, Error, Defense in advanced zones, Receiving a dangerous situation, Defense of goalkeeper (2) *
[51]	Constant, Receiving a dangerous situation, Defense closed to the own goal, Error, Keeper claim, High intensity confrontation, Mid-positioning defense with pressure, Defense in advanced zones, Keeper smother

* Two different factors have been given the same name.

## Data Availability

Not applicable.
